# High inter‐follicular spatial co‐localization of CD8+FOXP3+ with CD4+CD8+ cells predicts favorable outcome in follicular lymphoma

**DOI:** 10.1002/hon.3003

**Published:** 2022-04-28

**Authors:** Yeman B. Hagos, Ayse U. Akarca, Alan Ramsay, Riccardo L. Rossi, Sabine Pomplun, Victoria Ngai, Alessia Moioli, Andrea Gianatti, Christopher Mcnamara, Alessandro Rambaldi, Sergio A. Quezada, David Linch, Giuseppe Gritti, Yinyin Yuan, Teresa Marafioti

**Affiliations:** ^1^ Centre for Evolution and Cancer and Division of Molecular Pathology The Institute of Cancer Research London UK; ^2^ Cancer Institute University College London London UK; ^3^ Department of Histopathology University College Hospitals London London UK; ^4^ Bioinformatics Istituto Nazionale Genetica Molecolare Milan Italy; ^5^ Hematology Unit Ospedale Papa Giovanni XXIII Bergamo Italy; ^6^ Pathology Unit Ospedale Papa Giovanni XXIII Bergamo Italy; ^7^ Department of Haematology University College London Hospital London UK; ^8^ Department of Oncology and Hematology‐Oncology University of Milan Milan Italy; ^9^ Cancer Immunology Unit University College London Cancer Institute University College London London UK; ^10^ Research Department of Haematology University College London Cancer Institute University College London London UK; ^11^ Centre for Molecular Pathology Royal Marsden Hospital London UK

**Keywords:** CD4+CD8+, CD8+FOXP3+, deep learning, follicular lymphoma, image analysis, multispectral immunofluorescence

## Abstract

The spatial architecture of the lymphoid tissue in follicular lymphoma (FL) presents unique challenges to studying its immune microenvironment. We investigated the spatial interplay of T cells, macrophages, myeloid cells and natural killer T cells using multispectral immunofluorescence images of diagnostic biopsies of 32 patients. A deep learning‐based image analysis pipeline was tailored to the needs of follicular lymphoma spatial histology research, enabling the identification of different immune cells within and outside neoplastic follicles. We analyzed the density and spatial co‐localization of immune cells in the inter‐follicular and intra‐follicular regions of follicular lymphoma. Low inter‐follicular density of CD8+FOXP3+ cells and co‐localization of CD8+FOXP3+ with CD4+CD8+ cells were significantly associated with relapse (*p* = 0.0057 and *p* = 0.0019, respectively) and shorter time to progression after first‐line treatment (Logrank *p* = 0.0097 and log‐rank *p* = 0.0093, respectively). A low inter‐follicular density of CD8+FOXP3+ cells is associated with increased risk of relapse independent of follicular lymphoma international prognostic index (FLIPI) (*p* = 0.038, Hazard ratio (HR) = 0.42 [0.19, 0.95], but not independent of co‐localization of CD8+FOXP3+ with CD4+CD8+ cells (*p* = 0.43). Co‐localization of CD8+FOXP3+ with CD4+CD8+ cells is predictors of time to relapse independent of the FLIPI score and density of CD8+FOXP3+ cells (*p* = 0.027, HR = 0.0019 [7.19 × 10^−6^, 0.49], This suggests a potential role of inter‐follicular CD8+FOXP3+ and CD4+CD8+ cells in the disease progression of FL, warranting further validation on larger patient cohorts.

## 1 INTRODUCTION

In the western world, follicular lymphoma (FL) is the second most common subtype of non‐Hodgkin lymphoma, accounting for between 20% and 25% of cases.^1^, ^2^ The disease tends to follow an indolent remitting and relapsing course, with great individual variability. While patients achieving a sustained response to first‐line treatment show prolonged survival, those who fail to achieve a response or relapse early after the end of the therapy have an adverse outcome.^3^, ^4^, ^5^, ^6^ Early identification of refractory/early relapsing cases and investigation of the biological basis is currently a major challenge.^7^

The tumor microenvironment (TME) plays a key role in the clinical course of FL. Two immune response gene expression signatures, IR1 and IR2, were identified to be predictive of long and short survival, respectively in FL.^8^ The IR1 signature included genes encoding both T cells and macrophage molecules, whereas the IR2 comprised genes expressed in macrophages, dendritic cells or both. This and subsequent molecular studies,^8^, ^9^, ^10^ suggested the potential importance of immune surveillance in FL raising the possibility of novel immune approaches. The role of immune T cells,^11^, ^12^, ^13^, ^14^, ^15^, ^16^, ^17^, ^18^ macrophages,^14^, ^15^, ^19^, ^20^, ^21^ NK/T cells^22^, ^23^ and myeloid cells^24^, ^25^ were investigated in FL generating inconsistent results. These studies were conducted on a different cohort of patients who might have different characteristics and analyzed using different computational pipelines that could potentially hamper consistency and comparison of the prognostic power of the different immune cells groups. Moreover, the composition of the intra‐follicular areas, containing neoplastic cells, are distinct from the inter‐follicular areas. However, most of the previous studies considered the FL TME as one homogenous ecosystem. The pattern of immune infiltration in these two sites is predictive of outcome.^15^, ^16^, ^26^ Thus, investigating the spatial interaction of immune cells in the two regions could provide new insight into the biology of FL. However, no computational image analysis software tailored to these cell compartments are available.

Recently, deep learning has gained a surge of interest in digital pathology^27^ demonstrating its relevance to predict the diagnosis of several malignant diseases including Lymphoma.^28^, ^29^, ^30^ It has been shown that this technology also serves as a discovery tool to identify novel cell populations associated with tumor progression. Automated microscopy analysis is a more reliable approach to enumerate infiltrating cell populations but there has been limited use of deep‐learning analysis to study the microenvironment in FL.^31^, ^32^

Thus, we decided to use multispectral immunofluorescence (M‐IF) images containing 15 immune cell markers to (1) develop a deep learning‐based method to identify cell phenotypes, (2) develop cell distribution and spatial analysis pipeline tailored to the tissue compartments of FL, and (3) identify novel immune phenotypes associated with time to progression (TTP). Such an integrated, high throughput approach enabled us to identify the clinically relevant spatial distribution of immune cell subsets in the inter‐follicular area of FL TME.

## 2 MATERIALS AND METHODS

### 2.1 Cohort study

Patients diagnosed at Papa Giovanni XXIII Hospital (Bergamo, Italy) with grade I‐IIIa FL between 01‐Jan‐2006 and 31‐Dec‐2015, treated with standard R‐CHOP or R‐CVP and with the availability of the diagnostic surgical biopsy were eligible for this study. Clinical information of 39 patients was gathered from the electronic charts. The diagnosis of FL was confirmed by three haematopathologists (Teresa Marafioti [TM], Alan Ramsay [AR], and Sabine Pomplun [SP]); who reviewed independently the morphology assessed by using H&E staining. The relevant immunostaining evaluated included CD20, CD3, BCL‐2, BCL‐6, CD10, CD21, MIB‐1. All cases expressed BCL‐2, CD10 and BCL‐6 and no areas of diffuse growth pattern were present. The diagnosis of FL, followed the criteria of the revised fourth edition of the WHO classification of tumors of hematopoietic and lymphoid tissues. The exclusion criteria applied included: stage I disease, bendamustine therapy and rituximab maintenance. Seven cases were excluded of which six showed suboptimal tissue sections affecting staining, and the additional case had received bendamustine treatment. The final number of analyzed cases was 32. The study was approved by the Ethics Committee (approval number REG. 197/17) and performed in accordance with the ethical standards of the 1964 Helsinki declaration and its later amendments. All patients provided written informed consent. Time to progression, measured from diagnosis to relapse/progression time, was used as a clinical endpoint.

### 2.2 Antibodies panels and multispectral immunofluorescence

Two to four micron thick formalin‐fixed paraffin‐embedded tissue sections of 32 FL and 4 normal tonsils were subjected to M‐IF applying a series of antibodies (Supplementary Table 1) panels detailed below to study specific immune‐cell populations: (a) Immune T cells: CD4/CD8/FOXP3/PD‐1; (b) Tumor‐associated macrophages: CD68/CD163/CD206/PD‐L1; (c) Myeloid cells: CD8/CD11b/CD14/CD15 and (d) Natural killer T cells: CD8/Granzyme B (Granz B)/Granulysin/CD16/CD56.

Before data collection, experimental conditions (e.g., primary antibody dilution and time of incubation, order of antibodies immunostaining and fluorophores selection) of M‐IF staining were optimized and validated against singleplex chromogenic immunostaining protocols on consecutive sections of reactive tonsil and classical FL specimens retrieved from the diagnostic files of TM, AR and SP's institution. Staining was carried out on a Leica BOND RX automated immunostainer (Leica Microsystems, Milton Keynes, UK) using tyramide signal amplification (TSA)‐based Opal method (Opal 7‐Color Automation immunohistochemistry Kit, Akoya Biosciences, Marlborough, MA, USA; Catalog No. NEL811001 KT). SOPs for each antibodies panels has been compiled and can be provided upon request to the corresponding authors.

### 2.3 Statistical analysis

All statistical analyses were carried out using the Python programming language. All correlation values were measured using the non‐parametric Spearman test. The *p*‐values were computed using the two‐sided unpaired, non‐parametric Wilcoxon method, considering *p* < 0.05 as significant. To correct for multiple testing, we applied Benjamini‐Hochberg (BH) method. Time to progression was estimated using the Kaplan–Meier method and two‐tailed log‐rank test using Lifelines (v0.25.4) Python package.^33^ Multivariate Cox regression analysis was performed using the Lifelines (v0.25.4) Python package.^33^

### 2.4 Code availability

All methods and analyses were implemented in Python. For reproducibility and ease of sharing, the code and its dependencies are packed into a Docker container. The code runs on both local and high‐performance clusters. The code is accessible upon request to the corresponding author or the first author.

## 3 RESULTS

### 3.1 Patient clinical characteristics

**FIGURE 1:**
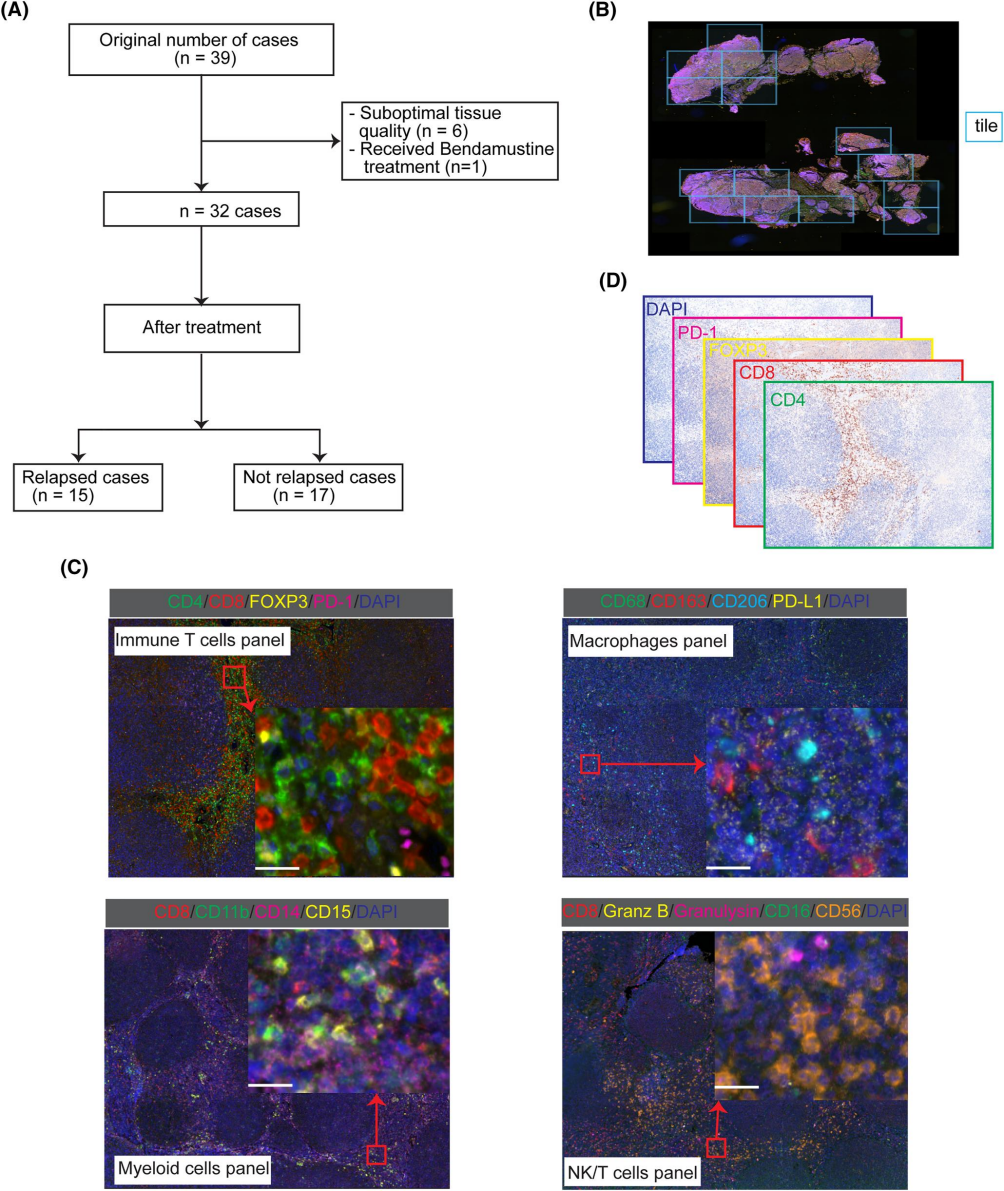
Details of the study cohort and M‐IF images. **A** Consolidated Standards of Reporting Trials (CONSORT) Diagram. **B** Illustrative image showing tile selection from a tissue section using VECTRA 3 platform. **C** Sample images from the four panels used in this study. Multispectral immunofluorescence images were acquired using VECTRA 3 with multiple markers in each panel. The scale bar is 10 μm. **D** Deconvoluted images for the immune T cell panel M‐IF image in **C.**

The clinical characteristics of the 32 patients included in this study are summarized in Table 1. The histological grade for all patients who relapsed varied between grades 1 and 2, but only one patient showed a focal grade 3A pattern. After a median follow‐up of 10.4 years (range 0.25–15.2 years), 23 patients remained alive. A total of nine deaths occurred and the causes were related to the progression of FL (3 cases); transformation to diffuse large B‐cell lymphoma (1 case); secondary cancer (1 case), unknown (occurring >10 years post‐treatment; 2 cases), complication of allogeneic stem‐cell transplantation (1 case) and acute hepatitis (1 case). Fifteen patients relapsed/progressed after a median of 2.83 years (range 0.6–14.8 years). The remaining 17 patients did not relapse after a median observation of 11.5 years (range 0.25–14.8 years; Figure 1A) with 4 deaths not related with disease progression.

**TABLE 1:** Patient characteristics

Clinical characteristics	All patients (N; %)
All patients	32 (100)
Age	
Median (range)	50.9 (30.5 – 77.9)
≥60	10 (31.3)
Gender	
Male	16 (50)
Ann Arbor stage	
III – IV	29 (90.6)
Bone marrow involvement	
Yes	21 (65.6)
FLIPI	
Low	3 (9.4)
Intermediate	16 (50)
High	13 (40.6)
TREATMENT	
R‐CHOP	26 (81.3)
R‐CVP	6 (18.7)

### 3.2 Deep learning for immune phenotyping in multispectral immunofluorescence images

The immune spatial phenotypes were investigated using four M‐IF panels for T cells, macrophages, myeloid cells, and natural killer *T* (NK/T) cells. The M‐IF images of diagnostic biopsies were acquired using the VECTRA 3 platform and the regions of interest defined as “tiles” were selected from different areas to capture the heterogeneity in the tissue section (Figure 1B–D).

**FIGURE 2:**
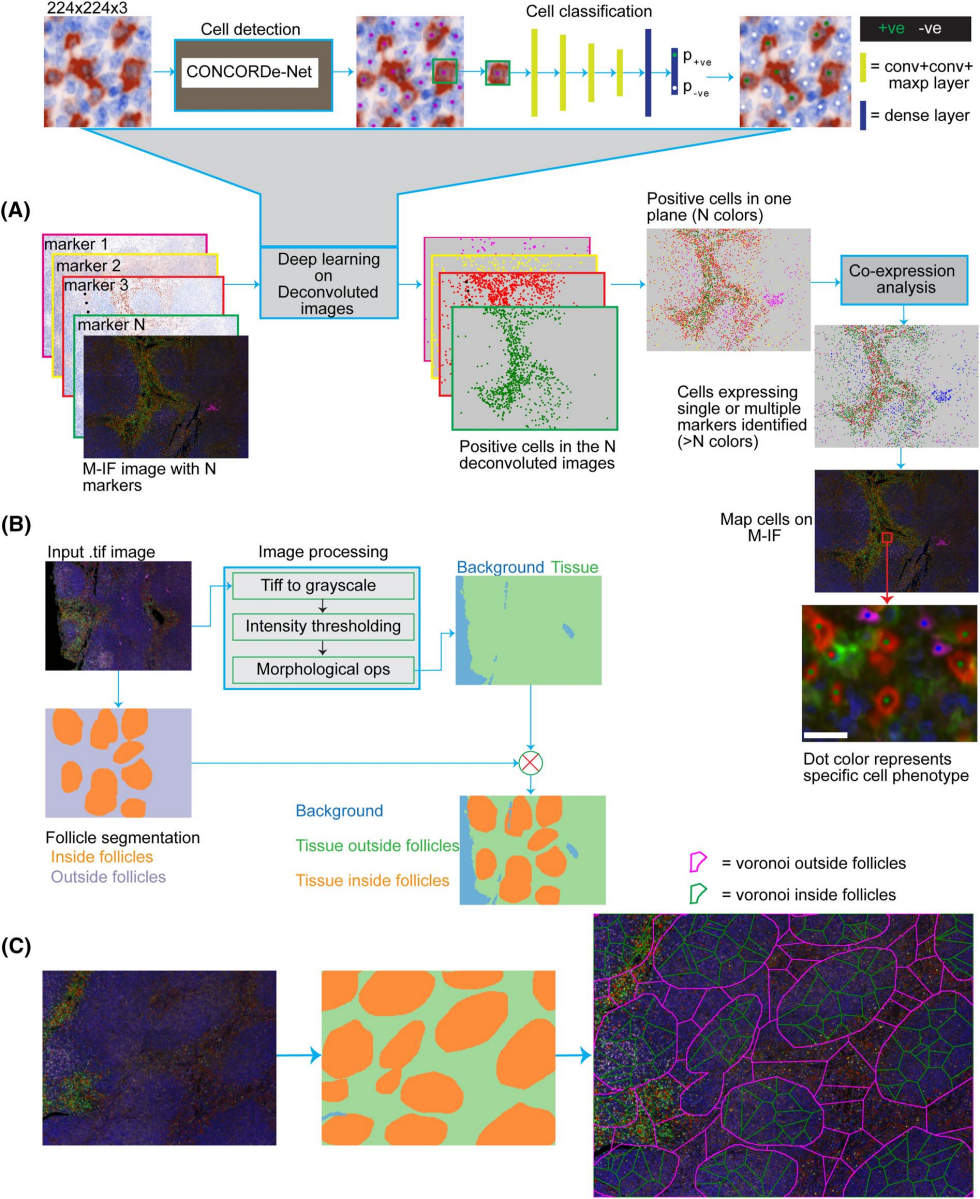
Computational deep learning and image processing pipelines. **A** Multi‐stage deep learning cell detection and classification pipeline for multispectral immunofluorescence (M‐IF). Cell detection and classification were applied to the *N* deconvoluted images. For cell detection, we applied CONCORDe‐Net (Methods in Supplementary Data). CONCORDe‐Net generates the x and y locations of the center of the nucleus of the cells. A patch centered on these cells’ location was extracted and fed to a VGG style convolutional neural network based cell classifier (conv = convolution, maxp = maxpooling layer). The classifier generates the probability of the input patch being positive (p_+ve_) and negative (p_‐ve_) for a marker. The class of the cell was assigned to positive (p_+ve_ ≥ p_‐ve_) or negative (p_+ve_ < p_‐ve_). This repeats for the *N* deconvoluted images. The positive cells from the *N* deconvoluted images were mapped onto one plane for co‐expression analysis. Then, cells were spatially mapped on M‐IF images. The bar indicates a 10 μm resolution. **B** A diagram of tissue and follicles segmentation. Follicles are manually segmented by an expert pathologist. Morphological ops = morphological operations. **C** Spatial Voronoi tessellation of within and outside follicles tissue compartments for Morisita‐horn index spatial analysis. Following the follicle segmentation in (B), the area within the follicle and outside follicle were divided into smaller polygons called “Voronoi”. Using location cells data in (A) cells can be mapped onto these polygons to apply spatial analysis

Though, M‐IF is a high‐throughput approach to characterize immune phenotypes landscape in tissue sections, the intermixing of colors deters accurate identification of cells and discernment of touching cells. In a computerized analysis of M‐IF, color (intensity) is the main discriminating feature between different cell phenotypes. Panels could have different colors (Figure 1C), and a supervised model trained on M‐IF data from one panel might not be generalized to another. However, irrespective of the number of markers/colors used in the M‐IF panels, the deconvoluted images in all panels have only brown (positive) and blue (DAPI, negative) colors (Figure 1D). This suggests that a model trained on deconvoluted images from one panel data could be generalized to the other panels. Thus, our newly developed deep learning image analysis pipeline systematically detects and classifies cells in M‐IF images from the deconvoluted images (Figure 2A and Methods in Supplementary Data).

### 3.3 Image and spatial analysis tailored to follicular lymphoma cellular compartments

The composition and spatial organization of immune cells in FL was analyzed in the intra‐follicular (within follicles) and inter‐follicular (outside follicles) regions, by developing a tissue and follicles segmentation pipeline (Figure 2B and Methods in Supplementary Data). This approach was designed to investigate whether distinct patterns of immune cell infiltrates in the two micro‐ecosystems represent a robust tool to predict clinical outcome.

To quantify cells spatial co‐localization and immune cell composition, we applied a Morisita‐Horn index^34^, ^35^, ^36^ to the regions within and outside the follicles separately (Figure 2C and Methods in Supplementary Data) and demonstrated differences between the two cellular compartments.

### 3.4 Deep learning models accurately map single cells in multispectral immunofluorescence images

**FIGURE 3:**
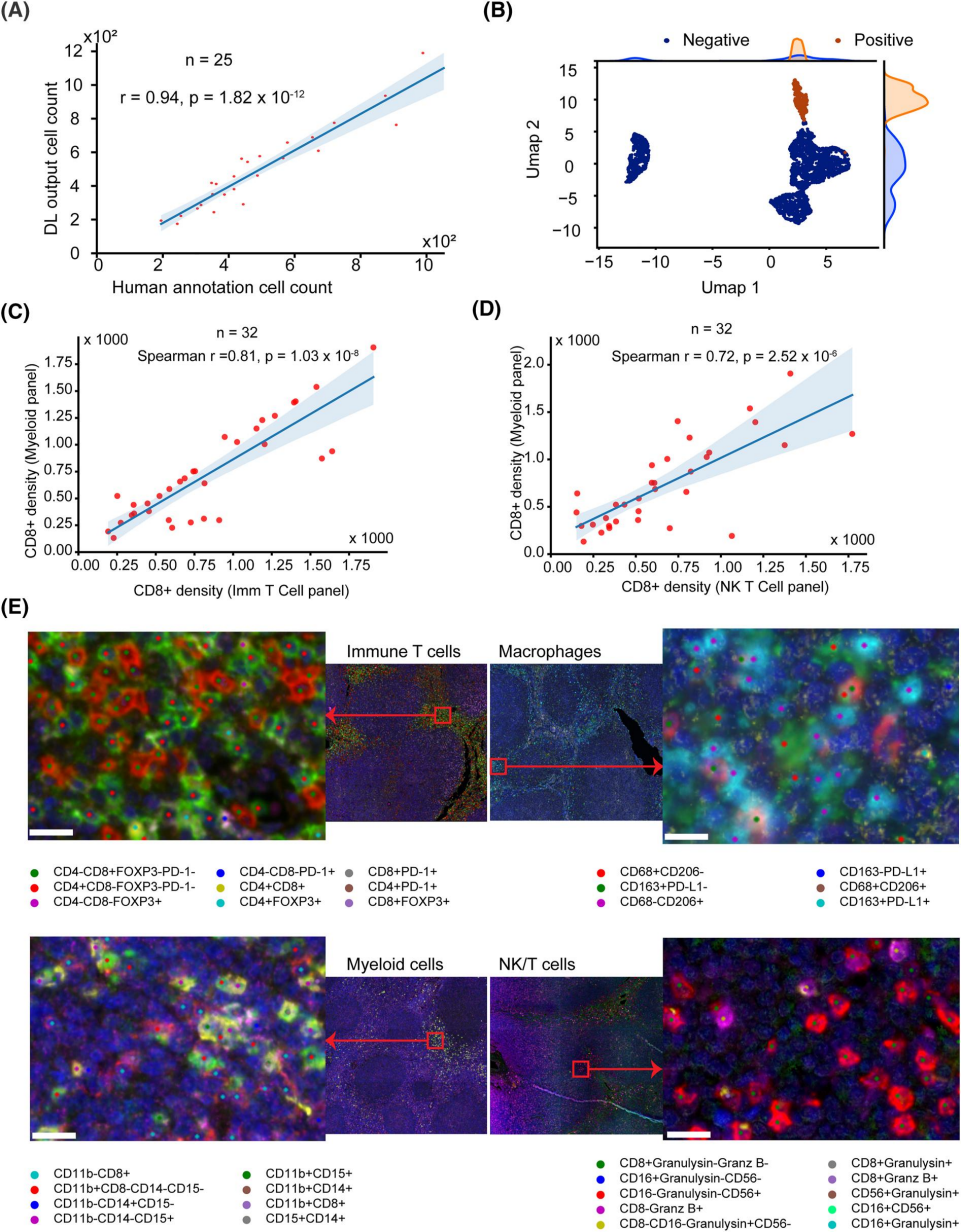
Evaluation and validation of deep learning models. **A** Correlation between the number of cells annotated by an expert and the number of cells detected by deep learning (DL). A dot represents a human‐annotated rectangular region, which contains around 450 cells. **B** Two‐dimensional representation of deep learned features after Uniform manifold approximation and projection (UMAP) dimensionality reduction along with their marginal distributions. Negative and positive classes represent cells negative and positive for a marker, respectively. **C, D** Deep learning model validation. The deep learning models were trained on immune T cell panel data. The trained model was then applied to all panels. The density of CD8+ cells (cells per 1000 μm^2^) across different panels was significantly correlated. A dot represents a sample or patient. All correlation values were computed using a non‐parametric Spearman correlation. **E** After detecting cells on the deconvoluted images, using the proposed co‐expression analysis (Methods in Supplementary Data), we were able to spatially map cells expressing single or multiple markers in all panels, which allows us to visually validate the deep learning models and co‐expression analysis on m‐IF images. The scale bar is 10 μm

To enable the automated detection and classification of diverse cell types in M‐IF images, we developed a deep learning pipeline (Figure 2A). The number of cells detected by the proposed deep learning method significantly correlated with the cells annotated by the expert pathologists (Spearman *r* = 0.94, *p* = 1.82 × 10^−12^ Figure 3A). Moreover, the proposed cell detection method achieved precision, recall and F1‐score values of 0.85, 0.86, and 0.86, respectively, on separately held test data (Supplementary Table 2). For cell classification, we obtained an area under the curve (AUC) of 0.995 (Supplementary Figure 1a) on separately held test data. Only 193 cells out of 10, 134 cells were wrongly classified (Supplementary Figure 1b). Visualization of the features learned by the convolutional neural network using uniform manifold approximation and projection (UMAP) dimensionality reduction demonstrated that cells of different classes are separated (Figure 3B and Supplementary Figure 1c).

The deep learning models were trained on the immune T cells panel data (Supplementary Table 2) and the models generalize to the other panels. The density of CD8+ cells across multiple panels is significantly correlated (Figure 3C–D and Supplementary Figure 1d) and cell classification AUC of 0.998 was obtained on single‐cell collected from the macrophages and NK/T cell panels, panels unseen during model training (Supplementary Figure 1e–f). The deep learning models and co‐expression analysis (Figure 2A and Methods in Supplementary Data) allowed us to identify different cell phenotypes in each panel (Figure 3E).

### 3.5 Decreased inter‐follicular CD8+FOXP3+ cells is associated with relapse

**FIGURE 4:**
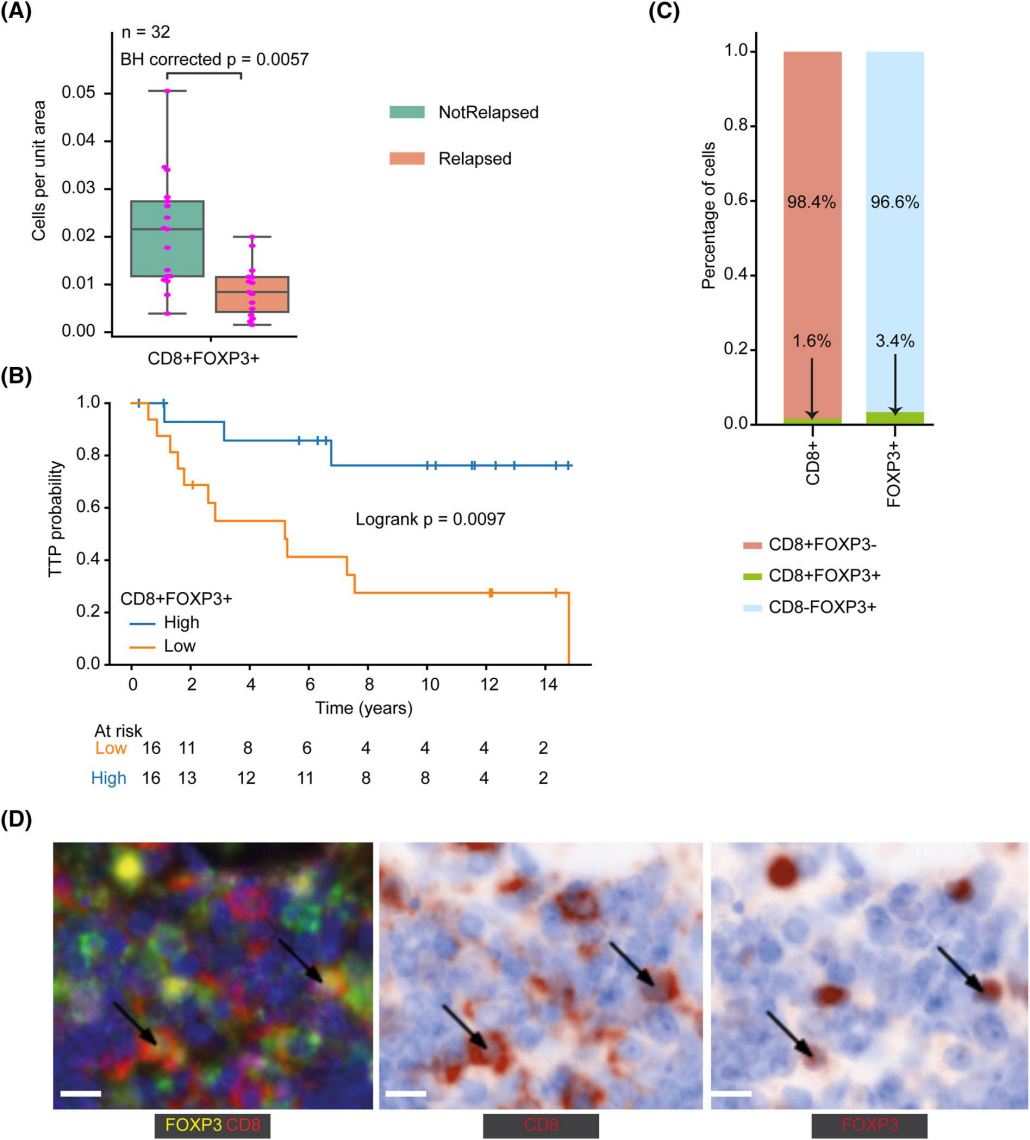
Prognostic cell subsets outside the neoplastic follicles. **A** Boxplot showing difference in density of CD8+FOXP3+ cells (cells/1000 μm^2^) outside follicles between relapsed (*n* = 15) and not relapsed (*n* = 17). **B** Kaplan‐Meier curves illustrating time to progression (TTP) of patients dichotomized using median CD8+FOXP3+ cells density outside follicles. **C** The percentage of CD8+ and FOXP3+ T cells expressing both CD8 and FOXP3 markers. **D** Sample illustrative image containing CD8+FOXP3+ cells. The arrows point to the center position of CD8+FOXP3+ cells detected by our deep learning method on M‐IF and deconvoluted images. The scale bar represents 10 μm. For statistical comparisons among groups, a two‐sided, nonparametric, unpaired, Wilcoxon signed‐rank test was used, unless stated otherwise. To correct for multiple testing, we applied Benjamini‐Hochberg (BH)

To identify prognostic cell types outside the neoplastic follicles, we first computed cell density. A significantly lower density of CD8+FOXP3+ cells outside the neoplastic follicles was found in diagnostic samples of patients who later relapsed, compared to those patients who did not relapse (BH corrected *p* = 0.0057, Figure 4A). Using Kaplan‐Meier estimates, increased CD8+FOXP3+ cells outside the neoplastic follicles was significantly associated with improved TTP using a median split (high 50% vs. low 50%: Logrank *p* = 0.0097, Figure 4B). The CD8+FOXP3+ cells accounted for 1.6% and 3.4% of CD8 marker and FOXP3 marker expressing immune cells, respectively (Figure 4C,D).

We also analyzed the association of the density of the remaining immune cells listed in Figure 3E in the inter‐follicular and intra‐follicular regions of FL with disease relapse and patient TTP (Supplementary Figures 2‐3 and Supplementary Tables 3‐4). However, none of these cells was significantly associated with disease relapse and patient TTP after applying multiple test corrections.

### 3.6 Clinical relevance of immune cell co‐localization

**FIGURE 5:**
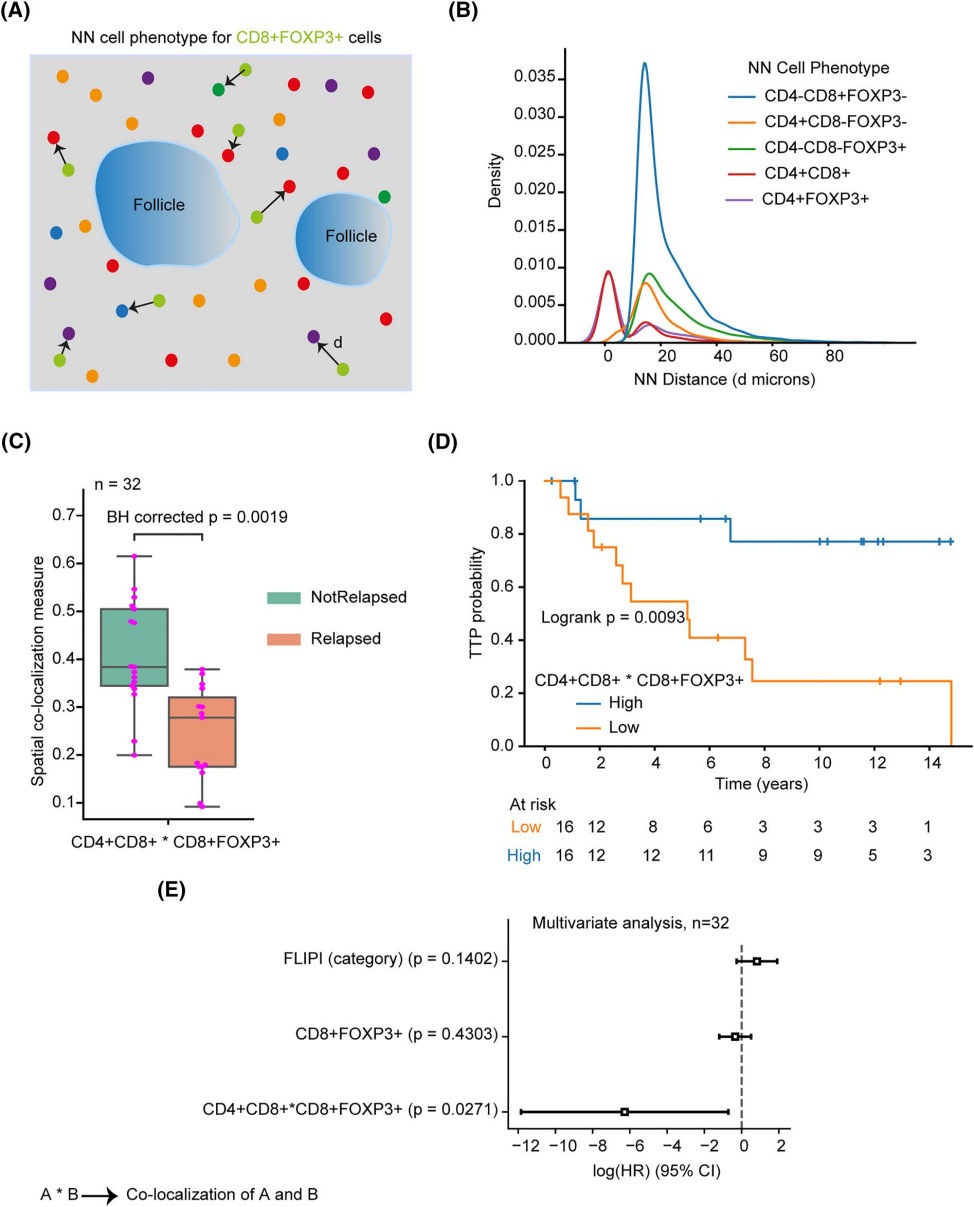
Prognostic spatial co‐localization of cell populations outside the neoplastic follicles. **A** Graphical representation of CD8+FOXP3+ cells nearest neighbor (NN) cells outside the neoplastic follicles. **B** The distribution of the distance of NN cells of different phenotypes. **C** Boxplot showing the difference in co‐localization of CD8+FOXP3+ with CD4+CD8+ cells outside follicles between relapsed (*n* = 15) and not relapsed (*n* = 17). **D** Kaplan‐Meier curves illustrating time to progression (TTP) of patients dichotomized using median co‐localization of CD8+FOXP3+ with CD4+CD8+ cells outside follicles. **E** Forest plots showing multivariate Cox regression analyses. Continuous values were used for the density and spatial localization parameters. Follicular lymphoma international prognostic index (**FLIPI**). For statistical comparisons among groups, a two‐sided, nonparametric, unpaired, Wilcoxon signed‐rank test was used, unless stated otherwise. To correct for multiple testing, we applied Benjamini‐Hochberg (BH)

To understand the spatial interaction of the inter‐follicular CD8+FOXP3+ cells with the other T cell subsets in the TME, we first explored their spatial neighborhood using nearest neighbor (NN) analysis (Figure 5A–B). For each CD8+FOXP3+ cell, we identified the NN cell phenotype and computed the distance in the tissue space (Figure 5A). In the inter‐follicular region, CD4+CD8+ and CD4+FOXP3+ NN cells tend to localize closer to CD8+FOXP3+ cells than other T cell subsets including CD4‐CD8+FOXP3‐, CD4+CD8‐FOXP3‐, and CD4‐CD8‐FOXP3+ cells.

We then asked if the co‐localization of these T cell subsets with CD8+FOXP3+ cells in the inter‐follicular region is associated with relapse and TTP. To quantify spatial co‐localization, we computed the Morisita‐Horn index, which increases in value if there is a high degree of spatial colocalization between two variables (Methods in Supplementary Data). The inter‐follicular co‐localization of CD8+FOXP3+ with CD4+FOXP3+ cells was not associated with relapse status (BH corrected *p* = 0.142, Supplementary Figure 4a) and patient TTP (Logrank *p* = 0.06, Supplementary Figure 4b) using Kaplan‐Meier estimates. However, lower degree of inter‐follicular co‐localization of CD8+FOXP3+ with CD4+CD8+ cells was associated with relapse (BH corrected *p* = 0.0019, Figure 5C). Using Kaplan‐Meier estimates, a higher degree of co‐localization of CD8+FOXP3+ with CD4+CD8+ cells was associated with longer TTP (Logrank *p* = 0.0093, Figure 5D).

### 3.7 Decreased inter‐follicular CD8+FOXP3+ cells density and spatial co‐localization of CD8+FOXP3+ with CD4+CD8+ cells are predictive of time to progression independent of follicular lymphoma international prognostic index

To investigate whether the inter‐follicular density of CD8+FOXP3+ and co‐localization of CD8+FOXP3+ with CD4+CD8+ are predictors of TTP independent of follicular lymphoma international prognostic index (FLIPI), we applied multivariate Cox regression analysis. For the regression analysis, continuous values of the density and spatial co‐localization scores were used. Tumors with low inter‐follicular co‐localization of CD8+FOXP3+ cells with CD4+CD8+ were at a significantly increased risk of relapse compared with tumors with a higher inter‐follicular co‐localization of these cell types (*p* = 0.027, Hazard ratio (HR) = 0.0019 [7.19 × 10^−6^, 0.49], Figure 5E) that was independent of FLIPI and density of CD8+FOXP3+ cells. Moreover, both inter‐follicular CD8+FOXP3+ cells density and co‐localization of CD8+FOXP3+ cells with CD4+CD8+ were not associated with FLIPI scores (Supplementary Figure 5a,b). However, there is a positive correlation between CD8+FOXP3+ density and co‐localization of CD8+FOXP3+ with CD4+CD8+ (Supplementary Figure 5c). Similarly, a low inter‐follicular density of CD8+FOXP3+ was associated with increased risk of relapse independent of FLIPI (*p* = 0.038, HR = 0.42 [0.19, 0.95], Supplementary Figure 5d), but not independent of co‐localization of CD8+FOXP3+ with CD4+FOXP3+ cells (*p* = 0.43, Figure 5E).

## 4 DISCUSSION

In this study, we developed a deep learning‐based image processing pipeline for M‐IF images to decipher the immune microenvironment in FL. To the best of our knowledge, this is the first study to analyze the distribution and spatial interaction of immune cells in the inter‐and intra‐follicular compartments of FL using high throughput M‐IF images and deep learning image analysis. In FL, the abundance and distribution of immune cells within and outside neoplastic follicles are distinct and heterogeneous,^26^ and thus the spatial interaction of the cells. The combination of M‐IF and deep learning‐based image analysis focused on FL compartments enabled us to identify novel prognostic cell populations and spatial patterns in FL.

Our study shows that in FL, the inter‐follicular CD8+FOXP3+ T cells are prognostic and positively correlate with patients TTP. Even though these cells account for a small fraction of CD8+ immune T cells in the TME of FL, it has been shown that rare cell types such as antigen‐specific T cells can play a crucial role in the development of cancer.^37^, ^38^ In 1970 Gershon and Kondo described a pool of CD8+ regulatory T cells which support tumorigenesis.^39^ This type of cells was subsequently described in prostate,^40^, ^41^ colon^42^ and non‐small cell lung cancer.^41^ In a mice model, Mayer et. al also showed that CD8+FOXP3+ cells have a light suppressive function.^43^ However, other studies support our results and showed that CD8+FOXP3+ cells have anti‐tumor cytotoxic activity. Using flow cytometry on mice treated with GMCSF secreting HER‐2/neu vaccine, CD8+FOXP3+ T cells were abundantly found in regressing and immunogenic tumors.^44^ CD8+FOXP3+ is a phenotype for anti‐tumor T cells, and such cells have a similar expression profile of activated T cells.^43^, ^44^

Triggering an effective immune response promotes the expansion of CD8+FOXP3+ lymphocytes.^44^ In a mice model, Le et al. demonstrated that CD4+ T cells promote the expansion of tumor‐specific T cells such as CD8+FOXP3+ cells by secreting stimulatory cytokines like IL‐2 and TGF‐β.^44^ Moreover, K. Y et al. showed that CD8+FOXP3+ T cells are immunosuppressive, but, their inhibitor function could be altered using Toll‐Like Receptor (TLR)‐8 signaling^40^, ^45^ suggesting this could be utilized by immunotherapeutic strategies in cancer.^40^, ^45^ Furthermore, it is reported that TLR signaling pathways interact with RCHOP immunochemotherapy that is used in FL.^46^ Further functional studies are needed to understand whether the CD8+FOXP3+ T cells in the TME of FL have an “innate” anti‐tumor function or this is modulated by exposure to the immunochemotherapy treatment.

To investigate the spatial interaction of inter‐follicular CD8+FOXP3+ cells with other cell types identified by our approach, we applied spatial co‐localization analysis (Figure 2C and Methods in Supplementary Data). We found that higher co‐localization of CD8+FOXP3+ cells with CD4+CD8+ in the inter‐follicular regions is associated with favorable TTP in FL. Previous studies described CD4+CD8+ cells as effector anti‐tumor T cells in a series of tumors for example, cutaneous T‐cell lymphoma,^47^, ^48^ nodular lymphocyte‐predominant Hodgkin lymphoma,^49^ and melanoma.^48^ The CD4+CD8+ T cells have a high IL‐2 cytokine secretion profile^47^ and interestingly, a high level of IL‐2 is reported to enhance the cytotoxic activity of CD8+ Tregs cells.^50^ These results suggest that CD4+CD8+ cells might have boosted the anti‐tumoral activity of CD8+FOXP3+ cells through an IL‐2 dependent pathway and thus resulting in a favorable patient outcome.

In this study, we showed that the combination of M‐IF, deep learning and regional spatial analysis is a promising strategy to identify novel immune cell phenotypes in FL that could stratify relapsed versus not relapsed FL patients, and predict TTP. However, our study has some limitations. Firstly, the data used to train and evaluate the deep learning models were generated from the same lab and device, which could impact the generalizability of the models. Secondly, the manual annotation of the neoplastic follicles is laborious, thus an automated deep learning methodology is a valuable development. Moreover, the small sample size and the highly variable indolent nature of FL^51^ could introduce bias to the results.

**FIGURE 6:**
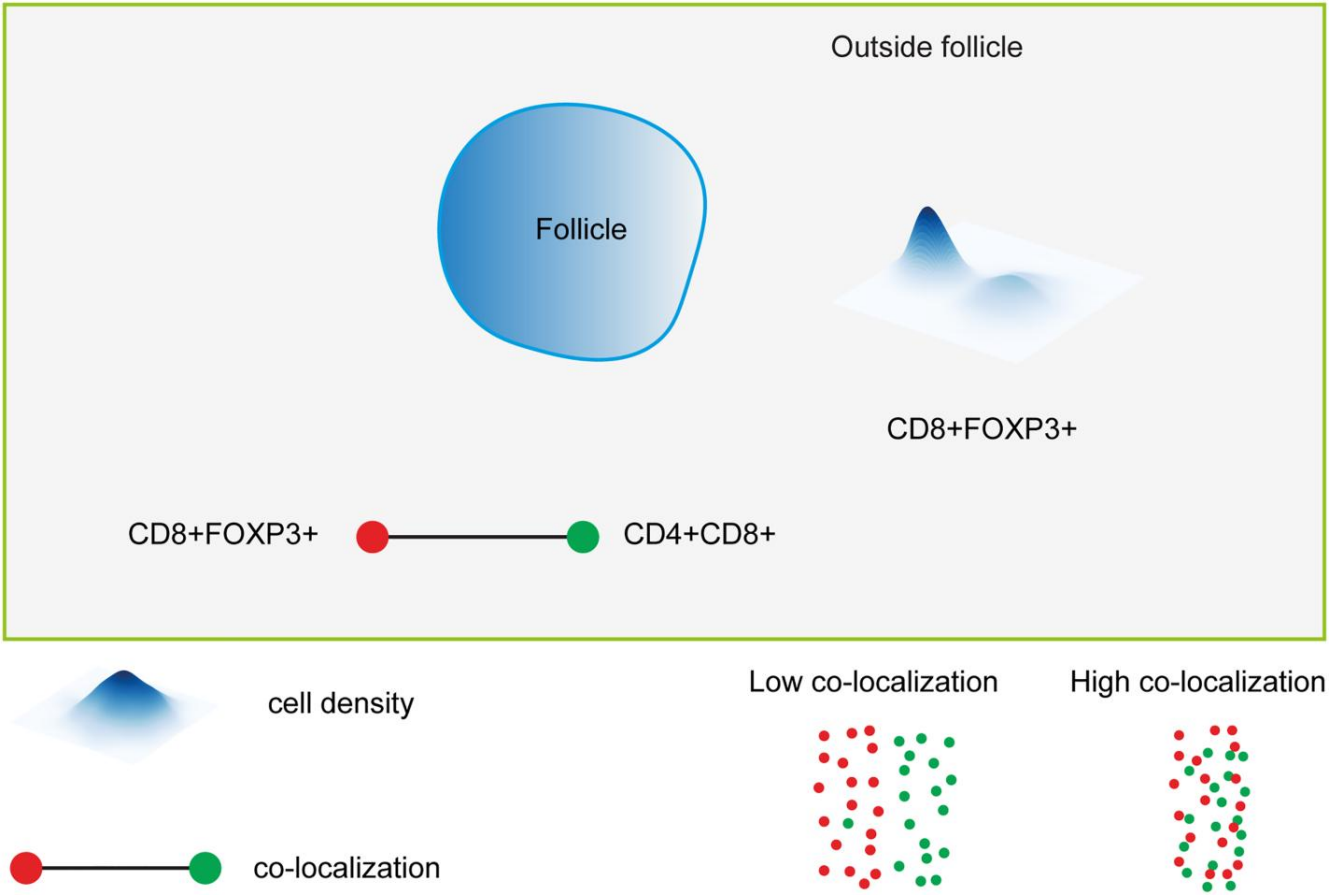
Graphical summary. The cartoon is a map of the two FL immune ecosystems: within and outside the neoplastic follicles indicating the clinically relevant cell co‐localization and the density of immune cell subsets

In summary, our study showed that low density of CD8+FOXP3+ cells or low co‐localization of CD8+FOXP3+ with CD4+CD8+ outside the neoplastic follicles (Figure 6) is associated with relapse and shorter TTP in FL patients treated with R‐CHOP or R‐CVP. The inter‐follicular density of CD8+FOXP3+ and co‐localization of CD8+FOXP3+ with CD4+CD8+ appear to be predictive of TTP independent of FLIPI score, and combining these features with FLIPI scores could improve prognostication. These findings require validation on a large cohort of FL patients treated with the same or different treatment regimens.

## CONFLICT OF INTEREST

The authors declare that they have no conflict of interest.

## AUTHOR’S CONTRIBUTIONS

Teresa Marafioti, Giuseppe Gritti and Yinyin Yuan conceived and designed the study; Yeman B Hagos developed the image analysis, deep learning pipelines, spatial analysis pipeline and performed statistical analysis. Ayse U Akarca optimized and digitized the M‐IF images. Giuseppe Gritti, Alessia Moioli and Andrea Gianatti collected the clinical cohort and provided the tissue sections. Alan Ramsay, Sabine Pomplun and Teresa Marafioti reviewed the patients and annotated single cells and follicules; Yeman B Hagos, Ayse U Akarca, Yinyin Yuan, Giuseppe Gritti and Teresa Marafioti wrote, edited and reviewed the manuscript. Riccardo L Rossi helped review the analyzed data. Alessandro Rambaldi and David Linch helped review and edit the manuscript. Victoria Ngai helped in annotating immune cells and follicles. Sergio A. Quezada and Alessandro Rambaldi contributed to reviewing the data and the manuscript. All authors gave final approval for publication. Christopher Mcnamara helped interpreting the data in the context of the clinical disease. Teresa Marafioti, Giuseppe Gritti and Yinyin Yuan take full responsibility for the work as a whole, including the study design, access to data and the decision to submit and publish the manuscript.

### PEER REVIEW

The peer review history for this article is available at https://publons.com/publon/10.1002/hon.3003.

## Supporting information

Supplementary Material

## Data Availability

The data that support the findings will be available upon request from the corresponding author.
