# Supplementary material for: High inter‐follicular spatial co‐localization of CD8+FOXP3+ with CD4+CD8+ cells predicts favorable outcome in follicular lymphoma
Source: Hematol Oncol. 2022 Apr 28;40(4):541–53. doi: 10.1002/hon.3003 (PMC10577604; doi:10.1002/hon.3003)
Supplement: Supplementary file 1 — Supplementary Material [file HON-40-541-s001.docx]

# Supplementary data

# Methods

## Image acquisition and cell annotation

All M-IF stained slides were scanned using the VECTRA 3 platform with data acquisition at 20× magnification. To train, test and validate the proposed deep learning models, we collected 41,695 single cells annotated on the deconvoluted images by two qualified pathologists (AR and TM) (Supplementary Table 2).

## Deep learning-based cell detection and classification on M-IF images

To detect cells on the deconvoluted images, we used cell count regularized convolutional neural networks (ConCORDe-Net) [1] which was implemented in Python (v.3.6) using the Tensorflow library (v2.2.0) [2]. The output of cell detection is the x and y position of the detected cells. These cells were classified into negative and positive classes using a custom-designed convolutional neural network (CNN). We then applied co-expression analysis to identify cells expressing single or multiple markers on the M-IF images (Figure 2a).

## Deep learning-based cell detection

Cell count regularized convolutional neural networks (ConCORDe-Net) [1] was used to detect cells. The model input is a 224x224x3 pixels image. ConCORDe-Net is implemented in Python (v.3.6) using TensorFlow library (v2.2.0) [2]. It was developed to detect cells in multiplex immunohistochemistry images and it outperformed state of the art cell detection models such as U-net [3] and MapDe [4] especially at detecting weakly stained and discerning touching cells. The training parameters and network architecture can be found in Hagos et. al. [1]. To discern weakly stained cells and touching cells, it utilizes cell count in the training data. Here, the model was trained from scratch using human annotations. The model has two outputs; predicted cell nucleus centre probability map image and cell count value. The predicted cell count was used to improve the predicted cell nucleus centre probability map by regularizing the training loss function.

## Deep learning-based cell classification

For cell classification, patches of size 20x20x3 pixels and 28x28x3 were extracted centred on the human annotations for nuclear and non-nuclear markers, respectively. We used smaller patch sizes for nuclear markers to minimize the effect of background noise. The number of single-cell annotations collected from non-nuclear markers was much higher than that of nuclear markers. To minimize the effect of this imbalance, we trained separate models for nuclear markers and non-nuclear markers.

InceptionV3[5] and VGG[6] are among the most commonly used classification CNNs [7]. Here, to minimize background and artefact in the training data, we proposed a smaller patch size compared to the input size of the original implementation of these CNNs. We custom-designed a shallow version of these CNNs because the input size is smaller than the input size used in the original implementation. The proposed shallow version architectures have a smaller number of parameters compared to the original implementation, and thus requires less memory and avoids overfitting during training. Normally, CNN based classification models have a feature learning section and classification section. The feature learning section is mainly composed of convolution and pooling layers, while the classification section is composed of dense and dropout layers. Feature learning section of InceptionV3 makes use of inception module, which has varying convolution kernel sizes and maxpooling layers. Feature learning section of VGG makes use of vgg module, which has a series of two convolution of same kernel size followed by maxpooling layer. To find an optimal depth classifier, we experimented with depth of 6 and 7 for both InceptionV3 and VGG architectures, and we used a final model with the best AUC score on a validation set.

The feature learning section starts with a convolution layer of 16 neurons and the number of neurons increases by 16 for every layer/module added. The classification section consists of two dense layers of {200, 2} neurons, with a dropout (rate = 0.3) layer in between. The two neurons in the output represent a positive and negative class. ReLU activation was used in all layers/modules, but softmax in the last layer to generate a probability. Model parameters were randomly initialized using uniform glorot [8], and optimized using Adam[9], learning rate 10^-4^ and categorical cross-entropy loss function. We trained the models for 500 epochs. All deep learning models were trained on The Institute of Cancer Research (ICR) high performance computing cluster.

The deep learning-based cell detection and classification enabled us to spatially map cells positive for all markers on the deconvoluted images. We then applied co-expression analysis to identify cells expressing single marker or co-expression multiple markers in the M-IF images.

## Identification of cells expressing single or multiple markers

After detecting positive cells on the deconvoluted, we identified cells expressing single or multiple markers as follows. Firstly, for a given tile, we mapped the location of the positive markers from its deconvoluted images onto a single plane. Then, to identify overlapping and non-overlapping markers, we computed a Euclidean distance between the markers in the image space. If the distance between detected markers on the deconvoluted images is less than 1.5µm, the markers are co-expressed on a cell. The distance value was empirically set.

## Deep learning model validation

The deep learning models were trained on data from an immune T cells panel (Supplementary Table 2) and applied to all four panels. Validation ensures the cell detection and classification models training on immune T cell data generalizes to the other panels. Validation was performed using two types of data. First, experts annotated 1367 single cells from macrophage and NK/T cells panels from CD206 and CD16 deconvoluted images (Supplementary Table 2). Model performance statistics were analysed using area under the curve (AUC) and confusion matrix. Secondly, CD8 is included in three out of four panels to verify the density of the CD8+ cells in these panels. The correlation of density of CD8+ cells between the panels was used to measure the generalizability of the deep learning models to the other panels. We expect a strong correlation of CD8+ cell density between the panels if the deep learning method is generalizable to all panels.

## Tissue and follicles segmentation

To segment the tissue from the background, we first converted the M-IF image into a grayscale image with an intensity range from 0 to 1. A threshold, *T* = 0.03 was applied to convert the grayscale image into a binary image. The value of *T* was optimized from the intensity profile of the grayscale images, and by visual inspection of the segmentation results. To smooth and fill holes in the binary image, we applied morphological closing (dilation followed by erosion) operations using disk structuring-element of radius 10 pixels (5µm). Mathematically, let *I_in_* be the input image, and *S* be the structuring element. The output image, *I_ou_*_t_ is computed as,

| $I_{out} =\left( I_{in}\bigoplus S \right)⊖S,$ | (3) |
| --- | --- |

where ⨁ and ⊖ denote the dilation and erosion, respectively.

Follicles were manually delineated by three accredited hematopathologists (AR, SP and TM). Two regions of interest were annotated for each FL tissue section: the region representing the neoplastic follicle (called within follicle) and the areas between neoplastic follicles (called outside follicles). Finally, by combining tissue and follicle segmentation, tissue areas within and outside follicles were identified.

## Cell density in the different tissue compartments of follicular lymphoma

Cell density is robust to variation in the amount of tissue compared to the abundance of cells since the earlier is the normalized by tissue area. Thus, we statistically compared cell densities instead of cell abundance between patient groups. To identify prognostic cell types within and outside the neoplastic follicles, we mapped cells to their respective region (within or outside follicle) and we computed cell density (cells per mm^2^) following the tissue and follicle segmentation results. For a given cell type, cell density within follicles is computed by dividing the number of cells by the tissue area (mm^2^) within follicles. A similar approach was applied for the outside follicles areas.

## Tessellation of FL cellular compartments and Morisita-Horn index

Morisita-Horn index is a measure of co-localization of two spatial variables used in ecological and immunological studies [10][11][12]. In FL, the regions within and outside follicles have distinct morphological and immune infiltration patterns [13][14][15][16]. Here, we hypothesized, the two tissue compartments differ in their cellular structure and thus we analysed the co-localization of cells in these regions separately. To compute the Morisita-Horn index, we first tessellated the tissue area into smaller regions. The most commonly used tessellation strategies are square and Voronoi tessellation [12]. We chose Voronoi tessellation because it mimics the natural distribution of spatial point patterns [12]. In Voronoi tessellation, an image is divided into a set of polygons using randomly selected seed points as a centre. The number of polygons (*N*) is determined by the tissue area as shown in Equation (6) for an image at 20x resolution [17]. To generate and visualize tessellation, we used Shapely[18] and scipy.spatial [19] Python packages.

| $N= \frac{\sqrt{Tissue area}}{48}$ | (6) |
| --- | --- |

We computed the Morisita-Horn index for the tissue within follicles as follows. Let *Z* be the number of tiles (T), collected from a given patient tissue section and let *Y* be the number of follicles (F) in *i^th^* tile, *T_i_*. Let *L* be the number of polygons in the *j^th^* follicle, *F_j_* obtained using Equation (6), which depends on the area of follicle *F_j_*. Since the tiles are non-overlapping, the polygons, *P,* used for spatial analysis is simply the set of combination of polygons generated from all *Z* tiles.

| *P* = $\left\{ P_{111},P_{112}, {\ldots, P}_{2jk}{, \ldots, P}_{3jk}, \ldots, P_{4jk}, \ldots,P_{ijk},\ldots, P_{ZYL} \right\},$ | (7) |
| --- | --- |

where *P_ijk_* is the *k^th^* polygon in follicle *F_j_* of Tile *T_i_*.

Then, we computed the number and proportion of each cell types in each polygon using the location and class labels of cells obtained from our proposed deep learning-based spatial mapping of cells in M-IF. Mathematically, let *C* be a set coordinates of cells within follicles in a given tile *T_i_*. Let *K* be a set of coordinates of randomly selected seed cells and let *(P_k_)_k є K_* be a seed cell for the *k^th^* polygon. A Voronoi region *R_k_* associated with *P_k_* consists of a set of cells closer to *P_k_* than *P_j_*, j≠k.

| $R_{k}=\left\{ c є C \right\vert d\left( c, P_{k} \right) \leq d\left( c, P_{j} \right), \forall j\neq k\}$, | (8) |
| --- | --- |

where *d* represents a Euclidean distance.

We then computed the spatial co-localization measure of Morisita-Horn index (m) for a pair of cell types, *c1* and *c2* as follows,

| $m =2 \frac{\sum_{k} X_{k}X_{k}^{'}}{\sum_{k} {(X_{k})}^{2}+ \sum_{k} {(X_{k}^{'})}^{2}} ,$ | (9) |
| --- | --- |

where *X_k_* and *X^’^_k_* are the proportion of *c1* and *c2* in the *k^th^* polygon, and 1≤ *k* ≤|*P*|, where |*P*| is the number of polygons within follicles in Equation (7). A similar procedure was followed for the region outside follicles. The value of *m* ranges from 0 (spatial segregation) to 1 (high spatial co-localization).

The value of *m* can slightly vary for multiple runs because seed points are chosen randomly and thus the polygons configuration. To incorporate this variation, we computed *m* for 12 iterations and the mean value was considered as a final spatial score.

## Tables

**Supplementary Table 1. Description of antibodies used in this study**

| **Molecule** | **Antibody type** | **Clone name** | **Dilution** | **Source** | **Opal fluorophore and dilution** |
| --- | --- | --- | --- | --- | --- |
| Anti-CD4 | Mouse Monoclonal | 4B12 | 1:50 | Leica Microsystems Ltd., Newcastle-upon-Tyne, UK | Opal-620 (1:150) |
| Anti-CD8 | Mouse Monoclonal | 4B11 | 1:200 | Leica Microsystems Ltd., Newcastle-upon-Tyne, UK | Opal-620 (1:150)  &  Opal-650 (1:200) |
| Anti-CD11b | Rabbit monoclonal | EP1345Y | 1:500 | Abcam Plc. Cambridge, UK | Opal-520 (1:100) |
| Anti-CD14 | Mouse Monoclonal | SP192 | 1:100 | Abcam Plc. Cambridge, UK | Opal-570 (1:100) |
| Anti-CD15 | Mouse Monoclonal | MMA | RTU | Leica Microsystems Ltd., Newcastle-upon-Tyne, UK | Opal-650 (1:100) |
| Anti-CD16 | Mouse Monoclonal | 2H7 | 1:40 | Leica Microsystems Ltd., Newcastle-upon-Tyne, UK | Opal-570 (1:100) |
| Anti-CD56 | Mouse Monoclonal | CD564 | RTU | Leica Microsystems Ltd., Newcastle-upon-Tyne, UK | Opal-520 (1:100) |
| Anti-CD68 | Mouse Monoclonal | PGM1 | 1:100 | Agilent Technologies LDA UK Ltd. Cheshire, UK | Opal-520 (1:100) |
| Anti-CD163 | Mouse Monoclonal | 10D6 | 1:200 | Leica Microsystems Ltd., Newcastle-upon-Tyne, UK | Opal-690 (1:100) |
| Anti-CD206 | Rabbit polyclonal | - | 1:250 | Abcam Plc. Cambridge, UK | Opal-620 (1:150) |
| Anti-Granulysin | Mouse Monoclonal | F-9 | 1:300 | Santa Cruz, Santa Cruz Biotechnology, Inc., Texas, U.S.A. | Opal-690 (1:100) |
| Anti-Granzyme B | Mouse Monoclonal | 11F1 | 1:80 | Leica Microsystems Ltd., Newcastle-upon-Tyne, UK | Opal-540 (1:100) |
| Anti-FOXP3 | Mouse Monoclonal | 236A/E3 | 1:2 | Kindly gifted by Dr G Roncador, CNIO, Madrid (Spain) | Opal-570 (1:150) |
| Anti-PD1 | Mouse Monoclonal | NAT 105/E3 | 1:350 | Abcam Plc. Cambridge, UK | Opal-540 (1:100) |
| Anti-PDL1 | Rabbit Monoclonal | 22C3 | RTU | Agilent Technologies LDA UK Ltd. Cheshire, UK | Opal-570 (1:100) |

**Supplementary Table 2. Distribution of human annotation collected from deconvoluted images belonging to negative and positive cell classes.**

| **Deconvoluted image** | **Negative cells** | **Positive cells** |
| --- | --- | --- |
| **Training (75%) and testing (25%) dataset from Immune T cells panel** | | |
| CD4 | 6 651 | 3 992 |
| CD8 | 5 254 | 985 |
| FOXP3 | 12 413 | 1 809 |
| PD-1 | 7 527 | 1 696 |
| **Validation data from NK/T cells and macrophages panels** | | |
| CD16 | 425 | 107 |
| CD206 | 594 | 241 |

**Supplementary Table 3. Statistical significance p value (between relapsed and not relapsed cases) and TTP logrank p values for density of cells outside follicles.** For statistical comparisons among groups, a two-sided, nonparametric, unpaired, Wilcoxon signed-rank test was used, unless stated otherwise. To correct for multiple testing, we applied Benjamini-Hochberg (BH).

| **Cell Name** | **P value** | **TTP logrank p value** |
| --- | --- | --- |
| CD16+/Granulysin-/CD56- | 0.23016 | 0.695645 |
| CD4-/CD8-/FOXP3+ | 0.233 | 0.281826 |
| CD11B+/CD14+ | 0.316867 | 0.058755 |
| CD15+/CD14+ | 0.316867 | 0.153487 |
| CD11B+/CD15+ | 0.316867 | 0.232623 |
| CD8-/CD11B+/CD14-/CD15- | 0.360238 | 0.675889 |
| CD11B-/CD14-/CD15+ | 0.469798 | 0.622003 |
| CD4+/FOXP3+ | 0.53056 | 0.833982 |
| CD11B-/CD14+/CD15- | 0.61019 | 0.866561 |
| CD8+/CD11B+ | 0.61019 | 0.253616 |
| CD163+/PDL1- | 0.79584 | 0.293668 |
| CD68-/CD206+ | 0.79584 | 0.397422 |
| CD68+/CD206+ | 0.89485 | 0.405931 |
| CD163+/PDL1+ | 0.89485 | 0.684397 |
| CD68+/CD206- | 0.89485 | 0.713578 |
| CD163-/PDL1+ | 0.89485 | 0.781852 |
| CD4-/CD8+/FOXP3- | 0.92479 | 0.456855 |
| CD56+/Granulysin+ | 0.92479 | 0.767382 |
| CD8+/Granulysin+ | 0.92479 | 0.807344 |
| CD4+/CD8+ | 0.92479 | 0.207722 |
| CD8-/Granulysin+/CD56-/CD16- | 0.92479 | 0.757035 |
| CD16+/Granulysin+ | 0.92479 | 0.844875 |
| CD16+/CD56+ | 0.92479 | 0.177508 |
| CD56+/Granulysin-/CD16- | 0.92479 | 0.212977 |
| CD4+/CD8-/FOXP3- | 0.92479 | 0.952659 |

**Supplementary Table 4. Statistical significance p value (between relapsed and not relapsed cases) and TTP logrank p values for density of cells inside follicles.** For statistical comparisons among groups, a two-sided, nonparametric, unpaired, Wilcoxon signed-rank test was used, unless stated otherwise. To correct for multiple testing, we applied Benjamini-Hochberg (BH).

| **Cell Name** | **P value** | **TTP logrank p value** |
| --- | --- | --- |
| CD8+/FOXP3+ | 0.14226 | 0.076275 |
| CD56+/Granulysin+ | 0.325973 | 0.259931 |
| CD16+/Granulysin-/CD56- | 0.325973 | 0.550402 |
| CD8-/Granulysin+/CD56-/CD16- | 0.325973 | 0.259931 |
| CD16+/CD56+ | 0.325973 | 0.310445 |
| CD4-/CD8-/FOXP3+ | 0.326 | 0.084855 |
| CD8-/CD11B+/CD14-/CD15- | 0.369798 | 0.766891 |
| CD8+/CD11B+ | 0.369798 | 0.763889 |
| CD11B+/CD15+ | 0.369798 | 0.763889 |
| CD11B+/CD14+ | 0.369798 | 0.550043 |
| CD15+/CD14+ | 0.369798 | 0.763889 |
| CD11B-/CD14-/CD15+ | 0.369798 | 0.763889 |
| CD8+/Granulysin+ | 0.371392 | 0.310445 |
| CD4-/CD8+/FOXP3- | 0.39792 | 0.309384 |
| CD4+/CD8+ | 0.39792 | 0.529019 |
| CD4+/FOXP3+ | 0.425844 | 0.495265 |
| CD68-/CD206+ | 0.503355 | 0.944195 |
| CD68+/CD206+ | 0.503355 | 0.606061 |
| CD163+/PDL1- | 0.503355 | 0.606061 |
| CD68+/CD206- | 0.503355 | 0.944195 |
| CD163-/PDL1+ | 0.52662 | 0.944195 |
| CD56+/Granulysin-/CD16- | 0.651385 | 0.310445 |
| CD163+/PDL1+ | 0.74822 | 0.944195 |
| CD16+/Granulysin+ | 0.8061 | 0.550402 |
| CD11B-/CD14+/CD15- | 0.86506 | 0.766891 |
| CD4+/CD8-/FOXP3- | 0.89485 | 0.921643 |

# Supplementary Figures


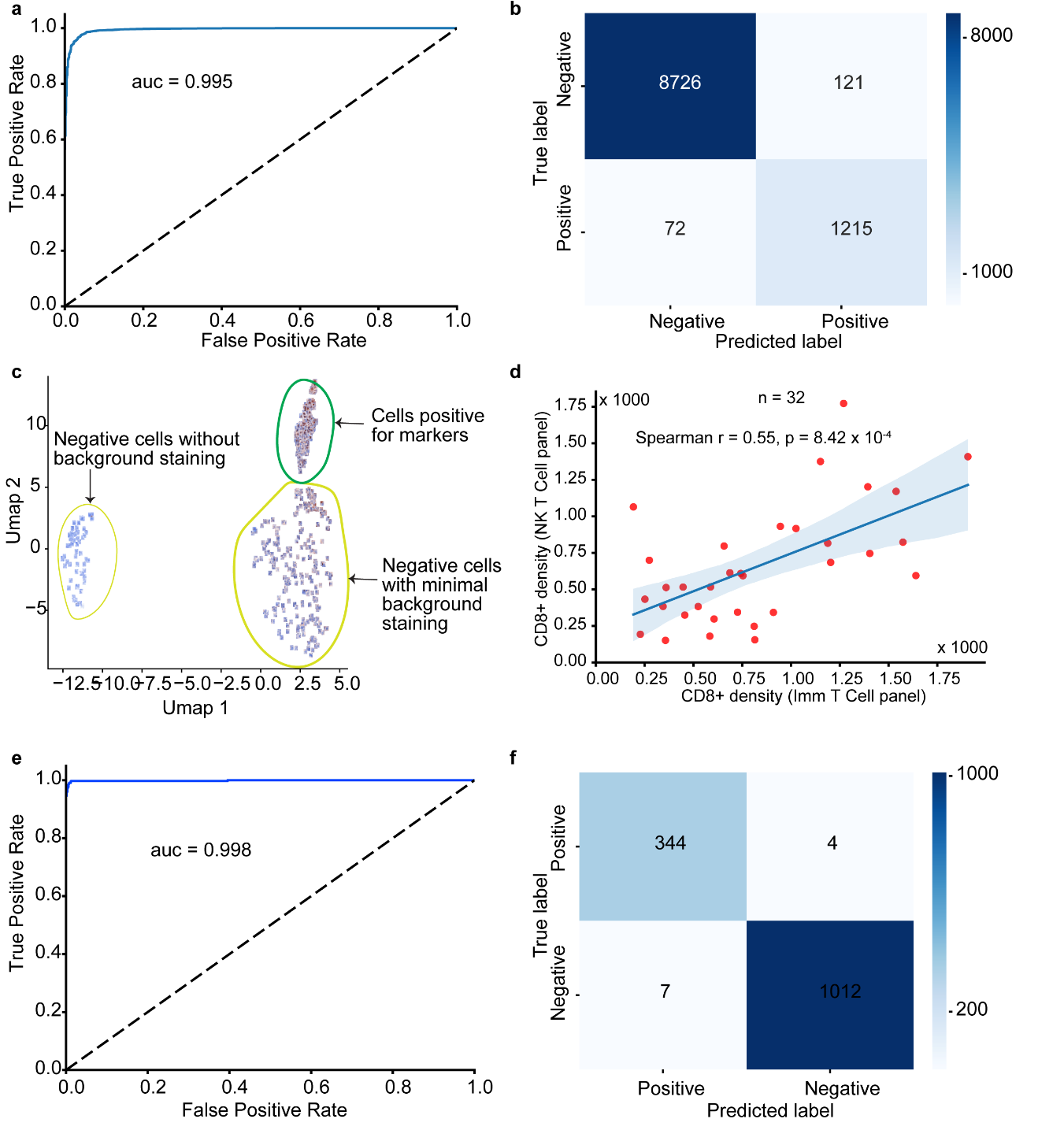
 ­­­­­­­

**Supplementary Figure 1. Deep learning models performance evaluation. a, b** Classifier performance evaluation using receiver operating characteristic curve (ROC) and area under the curve (AUC) (**a**) and confusion matrix (**b**) on a separately held test data. **c** Image scatters plot showing two-dimensional mapping sampled testing data after UMAP dimensionality reduction of deep learning features. **d** Spearman correlation of density of CD8+ cells. **e, f** Classifier performance evaluation using receiver ROC and AUC (**e**) and confusion matrix (**f**) on validation data collected from macrophages and NK/T cells panels.


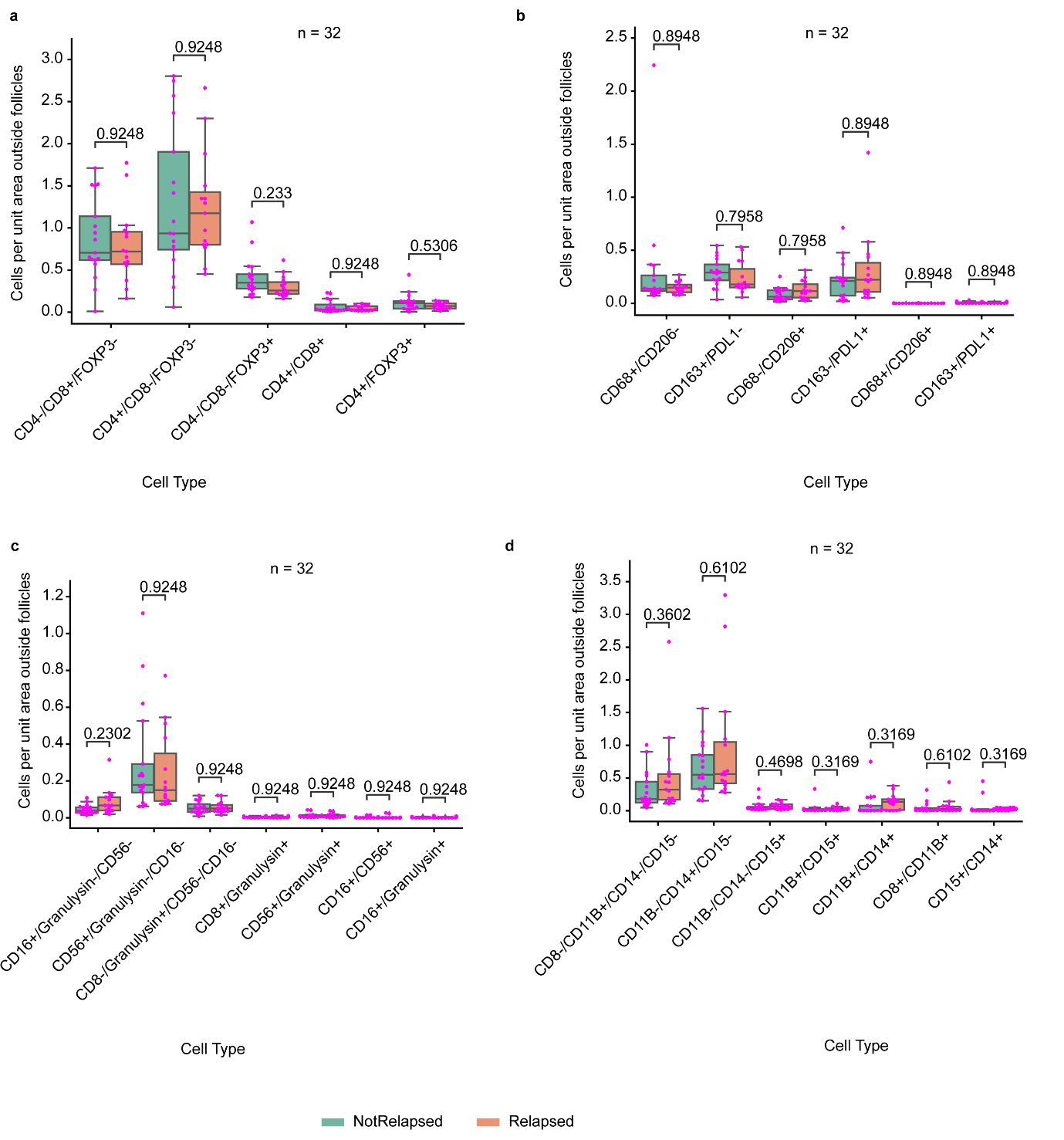


**Supplementary Figure 2. Distribution cell phenotypes outside follicles. a-f** Boxplot showing difference in density of cells (cells/ 1000µm^2^) outside follicles between relapsed (n = 15) and not relapsed (n = 17) cases. For statistical comparisons among groups, a two-sided, nonparametric, unpaired, Wilcoxon signed-rank test was used. To correct for multiple testing, we applied Benjamini-Hochberg (BH). All p values displayed are after multiple testing correction.


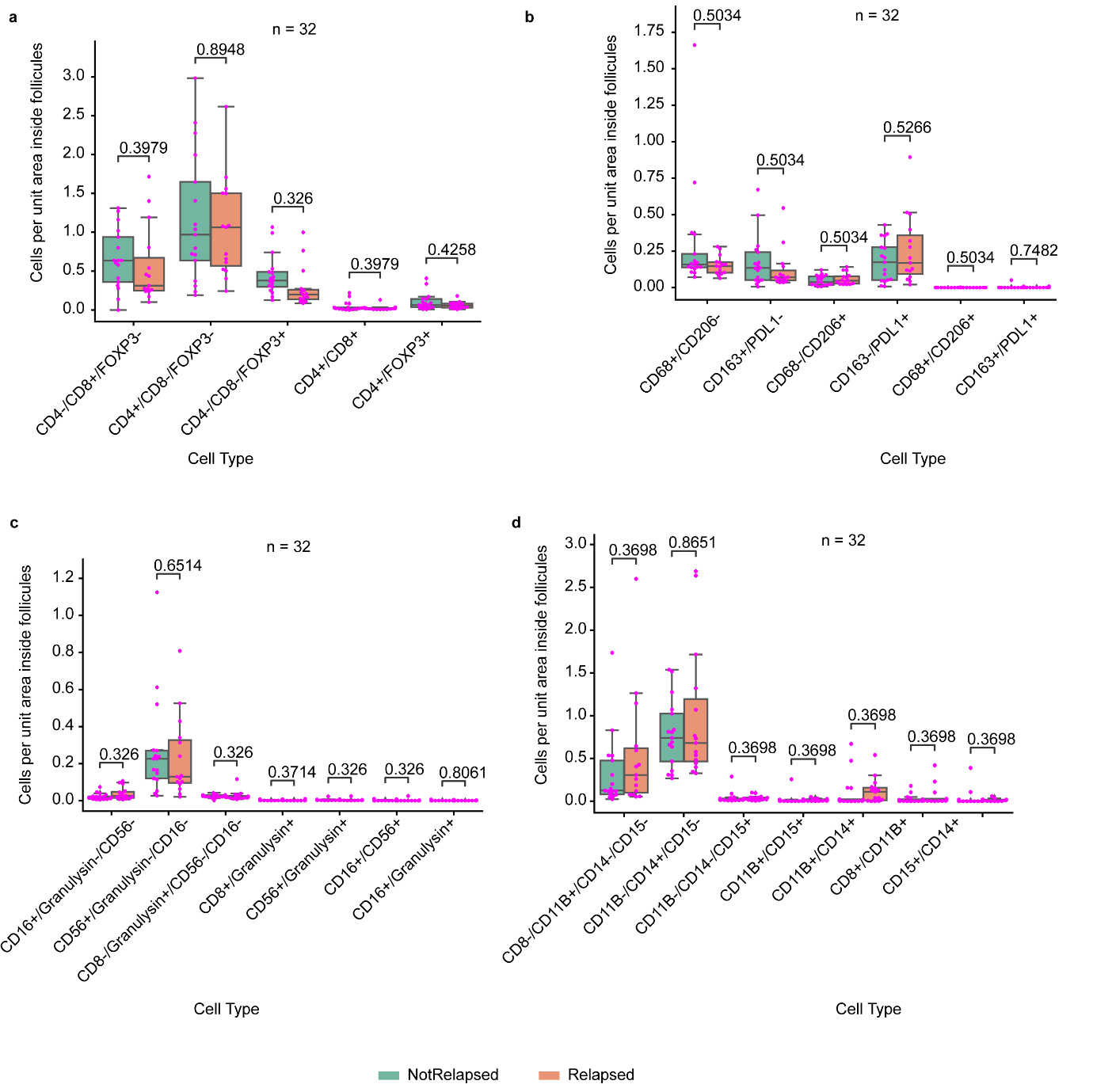


**Supplementary Figure 3. Distribution cell phenotypes inside follicles. a-f** Boxplot showing difference in density of cells (cells/ 1000µm^2^) inside follicles between relapsed (n = 15) and not relapsed (n = 17) cases. For statistical comparisons among groups, a two-sided, nonparametric, unpaired, Wilcoxon signed-rank test was used. To correct for multiple testing, we applied Benjamini-Hochberg (BH). All p values displayed are after multiple testing correction.


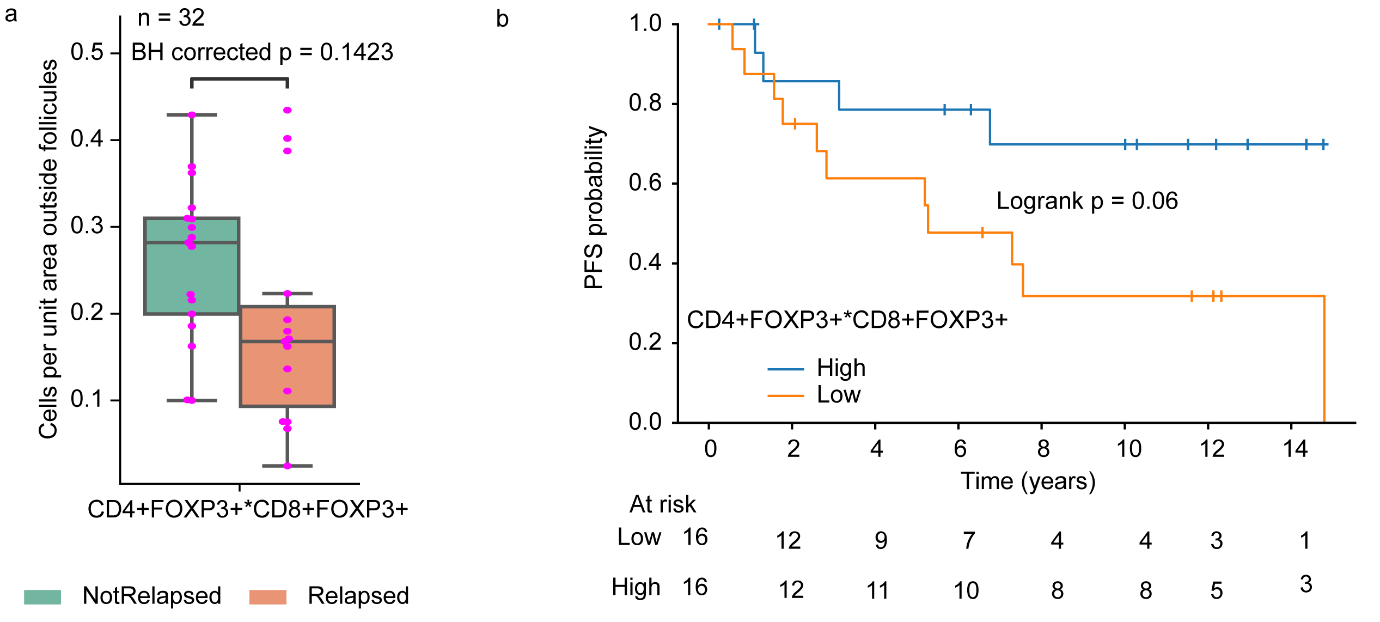


**Supplementary Figure 4. Co-localization of CD8+FOXP3+ with CD4+FOXP3+ outside follicle. a** Boxplot showing difference in co-localization of CD8+FOXP3+ with CD4+FOXP3+ cells outside follicels between relapsed (n = 15) and not relapsed (n = 17) cases. **d** Kaplan-Meier curves illustrating TTP of patients dichotomized using median co-localization of CD8+FOXP3+ with CD4+FOXP3+ cells outside follicles.

s
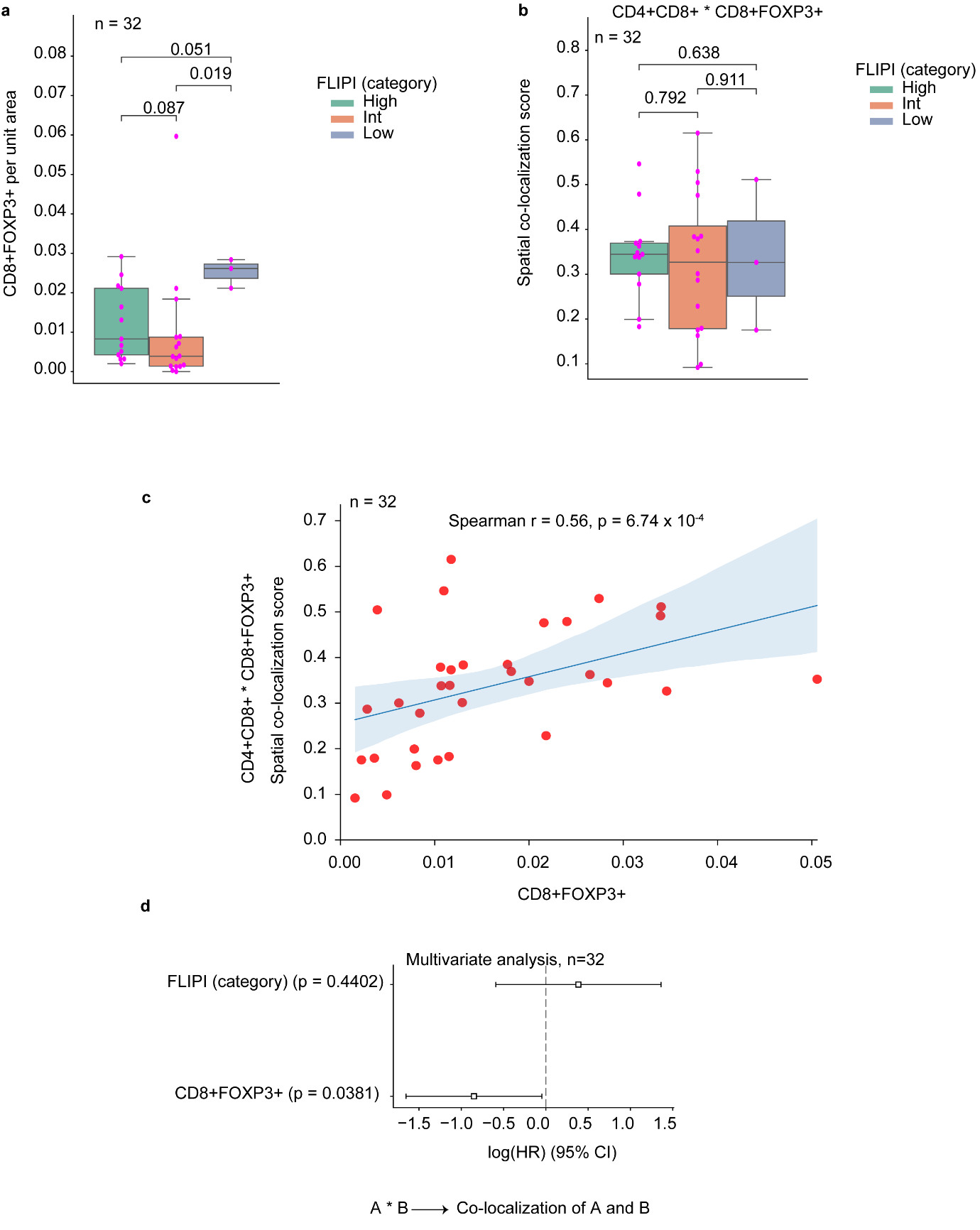


**Supplementary Figure 5. Association of inter-follicular CD8+FOXP3+ cell density with other standard scores. a** Boxplot showing difference interfollicular CD8+FOXP3+ cell density between the three FLIPI categories. **b** Boxplot showing difference interfollicular spatial score between the three FLIPI categories.  **c** Correlation between spatial score and density values. For statistical comparisons among groups, a two-sided, nonparametric, unpaired, Wilcoxon signed-rank test was used, unless stated otherwise. **d** Forest plots showing multivariate Cox regression analyses. Continuous values were used for the density parameter.  Follicular lymphoma international prognostic index (**FLIPI**).

## References

[1] Y. B. Hagos, P. L. Narayanan, A. U. Akarca, T. Marafioti, and Y. Yuan, “ConCORDe-net: Cell count regularized convolutional neural network for cell detection in multiplex immunohistochemistry images,” in *Lecture Notes in Computer Science (including subseries Lecture Notes in Artificial Intelligence and Lecture Notes in Bioinformatics)*, Oct. 2019, vol. 11764 LNCS, pp. 667–675, doi: 10.1007/978-3-030-32239-7_74.

[2] M. Abadi *et al.*, “TensorFlow: A system for large-scale machine learning.” 2016, Accessed: Mar. 05, 2021. [Online]. Available: https://research.google/pubs/pub45381/.

[3] O. Ronneberger, P. Fischer, and T. Brox, “U-net: Convolutional networks for biomedical image segmentation,” in *Lecture Notes in Computer Science (including subseries Lecture Notes in Artificial Intelligence and Lecture Notes in Bioinformatics)*, 2015, vol. 9351, pp. 234–241, doi: 10.1007/978-3-319-24574-4_28.

[4] S. E. A. Raza *et al.*, “Deconvolving convolutional neural network for cell detection,” in *Proceedings - International Symposium on Biomedical Imaging*, Apr. 2019, vol. 2019-April, pp. 891–894, doi: 10.1109/ISBI.2019.8759333.

[5] C. Szegedy, V. Vanhoucke, S. Ioffe, J. Shlens, and Z. Wojna, “Rethinking the Inception Architecture for Computer Vision,” in *Proceedings of the IEEE Computer Society Conference on Computer Vision and Pattern Recognition*, Dec. 2016, vol. 2016-Decem, pp. 2818–2826, doi: 10.1109/CVPR.2016.308.

[6] K. Simonyan and A. Zisserman, “Very deep convolutional networks for large-scale image recognition,” Sep. 2015, Accessed: Feb. 25, 2021. [Online]. Available: http://www.robots.ox.ac.uk/.

[7] M. Shu, “Deep learning for image classification on very small datasets Deep learning for image classification on very small datasets using transfer learning using transfer learning,” 2019. Accessed: Mar. 06, 2021. [Online]. Available: https://lib.dr.iastate.edu/creativecomponents.

[8] X. Glorot and Y. Bengio, “Understanding the difficulty of training deep feedforward neural networks,” JMLR Workshop and Conference Proceedings, Mar. 2010. Accessed: Feb. 25, 2021. [Online]. Available: http://www.iro.umontreal.

[9] D. P. Kingma and J. L. Ba, “Adam: A method for stochastic optimization,” Dec. 2015, Accessed: Feb. 25, 2021. [Online]. Available: https://arxiv.org/abs/1412.6980v9.

[10] A. Heindl, I. Sestak, K. Naidoo, J. Cuzick, M. Dowsett, and Y. Yuan, “Relevance of Spatial Heterogeneity of Immune Infiltration for Predicting Risk of Recurrence after Endocrine Therapy of ER+ Breast Cancer,” *J. Natl. Cancer Inst.*, vol. 110, no. 2, Feb. 2018, doi: 10.1093/jnci/djx137.

[11] C. C. Maley, K. Koelble, R. Natrajan, A. Aktipis, and Y. Yuan, “An ecological measure of immune-cancer colocalization as a prognostic factor for breast cancer,” *Breast Cancer Res.*, vol. 17, no. 1, p. 131, Sep. 2015, doi: 10.1186/s13058-015-0638-4.

[12] Y. Yuan, “Spatial heterogeneity in the tumor microenvironment,” *Cold Spring Harb. Perspect. Med.*, vol. 6, no. 8, Aug. 2016, doi: 10.1101/cshperspect.a026583.

[13] D. De Jong *et al.*, “Impact of the tumor microenvironment on prognosis in follicular lymphoma is dependent on specific treatment protocols,” *Haematologica*, vol. 94, no. 1, pp. 70–77, Jan. 2009, doi: 10.3324/haematol.13574.

[14] P. Farinha, A. Al-Tourah, K. Gill, R. Klasa, J. M. Connors, and R. D. Gascoyne, “The architectural pattern of FOXP3-positive T cells in follicular lymphoma is an independent predictor of survival and histologic transformation,” *Blood*, vol. 115, no. 2, pp. 289–295, Jan. 2010, doi: 10.1182/blood-2009-07-235598.

[15] B. E. Wahlin *et al.*, “A unifying microenvironment model in follicular lymphoma: Outcome is predicted by programmed death-1-positive, regulatory, cytotoxic, and helper T cells and macrophages,” *Clin. Cancer Res.*, vol. 16, no. 2, pp. 637–650, Jan. 2010, doi: 10.1158/1078-0432.CCR-09-2487.

[16] M. Saifi *et al.*, “High ratio of interfollicular CD8/FOXP3-positive regulatory T cells is associated with a high FLIPI index and poor overall survival in follicular lymphoma,” *Exp. Ther. Med.*, vol. 1, no. 6, pp. 933–938, Nov. 2010, doi: 10.3892/etm.2010.146.

[17] A. Heindl *et al.*, “Microenvironmental niche divergence shapes BRCA1-dysregulated ovarian cancer morphological plasticity,” *Nat. Commun.*, vol. 9, no. 1, pp. 1–14, Dec. 2018, doi: 10.1038/s41467-018-06130-3.

[18] Sean, Gillies and Others, “Shapely: manipulation and analysis of geometric objects.” 2007, [Online]. Available: https://github.com/Toblerity/Shapely.

[19] P. Virtanen *et al.*, “SciPy 1.0: fundamental algorithms for scientific computing in Python,” *Nat. Methods*, vol. 17, no. 3, pp. 261–272, Mar. 2020, doi: 10.1038/s41592-019-0686-2.
